# Effects of Individualized High‐Intensity Online Concurrent Exercise Guided by Autonomic Modulation on the Mental Health and Quality of Life of Breast Cancer Survivors

**DOI:** 10.1002/pon.70348

**Published:** 2025-12-03

**Authors:** Ana Myriam Lavín‐Pérez, Daniel Collado‐Mateo, Inés Nieto, Xián Mayo, Carmen Hinojo González, Ana de Juan Ferré, Alfonso Jiménez

**Affiliations:** ^1^ Sport Sciences Research Centre King Juan Carlos University Madrid Spain; ^2^ GO Fit LAB GO Fit Life Science and Technology, S.A. Madrid Spain; ^3^ Oncology Department Hospital Universitario Marqués de Valdecilla Santander Spain; ^4^ Instituto de Investigación Marqués de Valdecilla (IDIVAL) Santander Spain

**Keywords:** home‐based exercise, HRQoL, HRV, interval training, oncology, psychological factors, strength training

## Abstract

**Background:**

Exercise has been shown to improve mental well‐being and health‐related quality of life (HRQoL) in breast cancer survivors. However, there is no evidence on the effects of online interventions tailored using heart rate variability (HRV).

**Aims:**

This study analyzed the effects of online high‐intensity interval and strength training, guided daily by autonomic modulation, compared to pre‐planned moderate to high‐intensity concurrent training and control, on HRQoL, pain, fatigue, anxiety, depression, life satisfaction, self‐esteem, and fear of movement in breast cancer survivors.

**Methods:**

A 16‐week randomized controlled trial was conducted with 54 participants assigned to HRV‐guided exercise, pre‐planned exercise, or usual care. Participants trained aerobic and strength three times per week under real‐time online supervision. Intensity was adjusted based on HRV for the HRV‐guided group. HRQoL, anxiety, depression, life satisfaction, self‐esteem and fear of movement were assessed pre‐ and post‐intervention.

**Results:**

Significant time‐by‐group interactions were found for HRQoL functional domains (physical *p* = 0.004, role *p* = 0.005, emotional *p* = 0.013, social *p* = 0.001) and symptoms (fatigue *p* < 0.001, pain *p* < 0.001, dyspnea *p* = 0.004, insomnia *p* = 0.012, constipation *p* = 0.014), as well as depression (*p* < 0.001), anxiety (*p* = 0.002), life satisfaction (*p* = 0.003), self‐esteem (*p* = 0.024), and fear of movement (*p* = 0.015). HRV‐guided exercise led to greater improvements, while the control group worsened. Moreover, the exploratory analysis suggested a higher degree of interconnected changes in the HRV‐guided group, and that dyspnea and fatigue might be the variables most strongly connected with anxiety, depression, and functioning‐related variables.

**Conclusion:**

Individualized online concurrent exercise, especially autonomic modulation‐guided, improves HRQoL and mental health in breast cancer survivors, representing a promising personalized rehabilitation strategy.

## 1 Introduction

The number of breast cancer survivors is increasing [1], which can be attributed to improvements in early diagnosis and treatment effectiveness, as well as an overall rise in the number of cases [2]. This situation is alarming, with approximately 40.8% of women experiencing the disease from 2020 to 2024 [1]. After finishing hospital treatments, women who have breast cancer face a range of physical, clinical, and psychological challenges that greatly affect their mental well‐being and overall quality of life [3].

Women who have undergone primary surgery, chemotherapy, and radiation therapy carry psychological difficulties that they have to cope with. These difficulties can include distressing emotions such as anger, anxiety, despair, helplessness, fear of death, and even suicidal thoughts, among others [3]. The cumulative effects of long‐term treatments can exacerbate these difficulties, further impacting their mental health and survivorship [4]. Numerous studies have demonstrated the high incidence of anxiety and depression in these women, even years after the treatment process, and its relation with the risk of mortality [5]. Additionally, aspects such as self‐esteem and self‐concept are often damaged due to the drastic changes in breast anatomy or even the impact on libido as a side effect of the treatments [5]. Moreover, most detected cancers are hormone‐related, requiring ongoing treatment for at least 5 years after chemotherapy which often comes with limiting side effects, such as cancer‐related fatigue and joint pain [6]. The accumulation of all these psychological effects ends up affecting women's life satisfaction and their health‐related quality of life (HRQoL) [7], which decreases drastically in this population and is related to mortality [8].

Therefore, it is important to find strategies and therapies that help women in this process to improve their HRQoL in the short and long term. Exercise is emerging as one of the best tools to mitigate some of the mentioned side effects as well as to reduce the risk of sarcopenia, obesity, cancer recurrence, and mortality [9]. Recent literature has mainly focused on these physical and clinical aspects [10], but there are still many unanswered questions regarding its effectiveness on mental health. Meta‐analyses of the effect of exercise interventions on psychological parameters, such as life satisfaction, depression, anxiety, fear of movement, self‐esteem and HRQoL, are highly heterogeneous in the type of exercise employed, the intensity, or even in the way they are carried out [11, 12].

Regarding the type, intensity, and frequency of exercise, there is a consensus in recommending a combination of aerobic and resistance training performed at moderate‐to‐vigorous intensities, typically around 60%–80% HRmax for aerobic exercise and 65%–85% 1RM for resistance training, at a frequency of 2–3 sessions per week to improve HRQoL, physical function, and psychological outcomes such as depression and anxiety [11, 13, 14]. These guidelines also highlight some variability in the recommended volume of resistance training depending on the target outcomes, generally ranging from 2 to 3 sets per exercise and 8 to 15 repetitions, reflecting heterogeneous dose and the need to adapt the workload according to patients' needs [13, 14]. However, when analyzing fatigue intensity, general recommendations decline to 65% of HRmax and 60% of 1RM (RPE ≈ 12) [13, 14]. In contrast, recent studies suggest that higher resistance loads (≥ 70% 1RM) can also be safe and effective in this population, potentially leading to greater gains in muscle strength and reductions in fatigue [15]. So, there is controversy regarding the implementation of high intensity in women with breast cancer who are experiencing the effects of fatigue or joint pain [6]. This variability indicates that a single, universal prescription may not be optimal for all breast patients, especially given the heterogeneity in treatment side effects, fatigue levels, and recovery capacity. In this context, individualized prescription strategies that account for day‐to‐day physiological fluctuations may be particularly useful to tailor intensity, ensuring safety while maximizing the benefits of exercise. Therefore, an individualized intervention based on objective parameters related to exercise recovery, such as heart rate variability (HRV), could be beneficial in prescribing high‐intensity exercise, particularly by examining the relationship between this variable and depression, anxiety, fatigue, and pain perception [16].

Concerning the mode of exercise implementation, telemedicine, home‐based, or digital interventions are gaining increasing importance and frequency thanks to the development of technologies [17]. However, many of these exercise programs have been based on physical activity recommendations or low or moderate‐intensity exercise, in contrast to applying the intensities and weights more commonly used in face‐to‐face training [17]. In order to get similar benefits, remote training should have similar characteristics to face‐to‐face exercise. Finally, supervision is highly relevant for achieving a greater sense of security, providing corrections, promoting social support, and exercising adherence [18, 19]. Online and remote exercise programs can be as effective as face‐to‐face programs by including real‐time connections between trainers and patients, which allows trainers to provide instructions and corrections to participants.

Currently, there is limited research on online exercise programs for breast cancer patients. The few existing studies included supervised aerobic and/or strength training [20, 21], but did not prescribe training according to patients' baseline levels. Additionally, technology can enhance the personalization of exercise programs by adjusting daily routines based on objective physiological imbalances. One promising tool, as mentioned above, is the daily prescription of exercises based on parasympathetic modulation, a technique previously used in sports performance [22]. This approach may help to further individualize patients' training and achieve significant mental and psychological benefits. Thus, the present research aimed to analyze the effects of an online high‐intensity interval and strength exercise program, prescribed based on daily parasympathetic modulation, compared to pre‐planned exercise of moderate to high intensity and usual care in HRQoL, pain, fatigue, anxiety, depression, life satisfaction, self‐esteem, and fear of movement in breast cancer survivors.

## 2 Methods

### 2.1 Study Design

This randomized controlled trial was registered on ClinicalTrials.gov (ID: NCT05040867), recognized by the WHO and ICMJE. The study adhered to the 2014 Declaration of Helsinki and received ethical approval from Universidad Rey Juan Carlos (No. 1901202103121) and Hospital Universitario Marqués de Valdecilla (IDIVAL, No. 42//2021). Moreover, the study was designed and reported in accordance with the CONSORT (Consolidated Standards of Reporting Trials) guidelines. All participants were informed of the study's purpose, potential risks, and benefits, and provided written informed consent prior to participation.

### 2.2 Participants

Recruitment occurred in two phases. First, the oncology team at Marqués de Valdecilla University Hospital identified eligible women aged 18–65 with luminal or triple‐negative breast cancer who had completed chemotherapy and radiotherapy 1–5 months prior, minimizing cardiotoxicity risks. Exclusion criteria included scheduled surgeries, metastases, prior cancers, and HER2+ status.

In the second phase, the research team contacted candidates. Those without access to a smartphone with internet, incompatible schedules, or injuries preventing exercise were excluded. All eligible participants underwent to an echocardiogram to examine severe heart conditions previously undiagnosed before random and blind assignment to one of three groups: HRV‐guided high‐intensity exercise, pre‐planned moderate‐to‐high‐intensity exercise, or usual care. Participants were enrolled by the medical team and allocated to intervention groups based on a predetermined randomization sequence, following the order in which they completed radiotherapy. The sample size was calculated using G*Power for a repeated measures ANOVA (3 groups, 2 time points), estimating a moderate effect size (*f* = 0.25) based on Kotte, Bolam [23] with *α* = 0.05 and power 0.80, resulting in 16 participants per group. To compensate for possible losses, 68 women were recruited.

The final sample had a mean age of 50.3 years (SD = 8). Cancer types included luminal B (44.44%), luminal A (37.04%), and triple‐negative (18.52%). Group characteristics are detailed in Table 1.

**TABLE 1:** Participants' characteristics.

	HRV‐guided exercise group	Pre‐planned exercise group	Control group	*p*‐value
Sample size	18	18	18	
Nationality (%)				
European	100	100	84.61	0.985
South American	0	0	5.18	0.859
Mean age (SD)	50.56 (7.56)	48.94 (9.27)	51.39 (7.19)	0.656
Type of breast cancer (%):				
Luminal phenotype A	38.89	33.33	38.89	0.928
Luminal B phenotype	38.89	50.00	44.44	0.931
Triple negative	22.22	16.67	16.67	0.89

Abbreviations: HRV: Heart rate variability; SD: Standard deviation.

### 2.3 Procedure

Participants were randomized into three groups. Two of them performed online supervised exercises with intensity guided by daily individual HRV (HRVG) or with pre‐planned exercise intensity (PEG) and the other one continued with usual care.

#### 2.3.1 Online and Supervised Concurrent Exercise Programs

Participants trained 3 days per week for 16 weeks, with each session lasting 60–70 min and conducted by a certified exercise professional specialized in oncology populations. Equipment necessary for the exercises was provided to each participant during the pre‐intervention evaluation. All sessions were supervised and conducted online. Participants connected via video call with an exercise professional and other participants, seeing as well real‐time heart rate, monitored by MZ‐Remote platform. Intensity adjustments were made based on heart rate for cardiovascular exercises and weightlifting load for strength exercises. Posture and technique were monitored and corrected during sessions.

**FIGURE 1:**
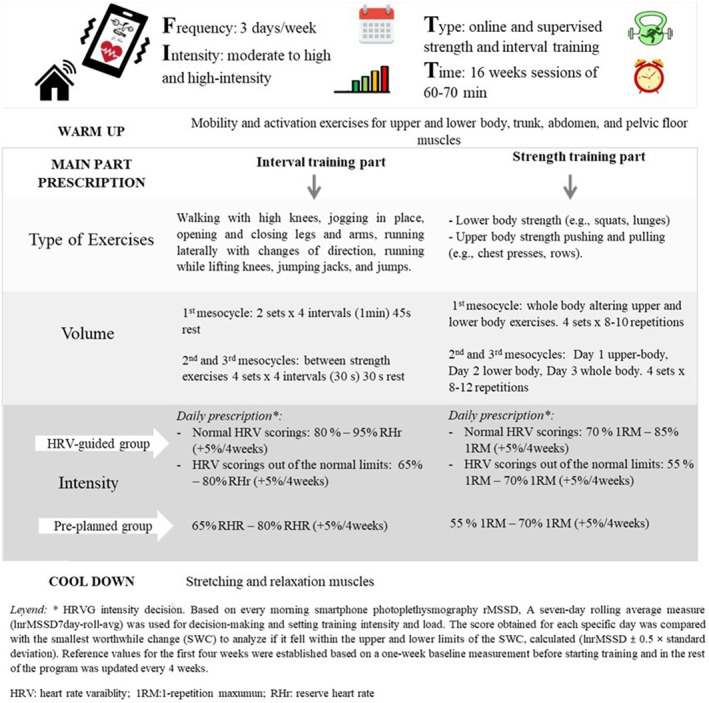
Online supervised concurrent exercise programs of the HRV‐guided group and the pre‐planned group.

Sessions were structured into three phases: warm‐up, main exercise, and cool‐down. The warm‐up lasted 5–10 min and included mobility and activation exercises for upper and lower body, trunk, abdomen, and pelvic floor muscles to prepare participants for the main exercise. The main phase lasted 45–50 min and included interval and strength training with increasing technical and biomechanical difficulty in each session. Finally, the cool‐down phase lasted around 5 min and involved stretching muscles, focusing on relaxation, and muscle lengthening during exhalation. All phases are explained in Figure 1.

In both exercise groups, intensity was prescribed according to the values achieved in the initial evaluation. However, the way of prescription was different between groups. In the PEG, the exercise prescription was prefixed following a blocked progression, whereas in the HRVG, the intensity varied based on individual daily HRV. This way of tailoring exercise allowed the trainer to adjust the training load according to the individual's physiological stress (rMSSD) measured by an HRV photoplethysmography. More detailed information on the exercise programs is presented in Figure 1 and explained elsewhere [24].

#### 2.3.2 Usual Care

The control group continued with the usual care including the treatments and without any exercise restriction.

### 2.4 Instruments and Variables

Evaluations took place 2 weeks before the intervention and immediately after the 16‐week program. All three groups completed self‐administered questionnaires. Sociodemographic data, including age, country of origin, and clinical information related to cancer diagnosis and treatment, were also collected.

#### 2.4.1 Measurement of Health‐Related Quality of Life

To assess HRQoL, participants were asked to complete the EORTC QLQ‐C30 questionnaire [25], designed to evaluate functionality (physical, role, emotional, cognitive, and social functioning), cancer‐related symptoms (fatigue, nausea and vomiting, pain, dyspnea, insomnia, loss of appetite, constipation, diarrhea, and financial difficulties), and overall health status [26]. The questionnaire uses a temporal scope of 1 week and a 4‐point Likert scale, ranging from “not at all” to “very much.”, except for the two items evaluating overall health status, which range from 1 to 7.

Following the manual, scores were transformed to a 0–100 scale, where higher scores indicate stronger responses [26]. On functional and global health status/quality of life scales, higher scores reflect better functioning or well‐being. In contrast, higher scores on symptom scales indicate greater symptom severity or problems [25].

#### 2.4.2 Measurement of Anxiety and Depression

Anxiety and depression were assessed using the Hospital Anxiety and Depression Scale (HADS) following established guidelines [27]. The 14‐item scale evaluates symptoms over the past week, with odd‐numbered items assessing anxiety (HADS‐A) and even‐numbered items assessing depression (HADS‐D). Each item is rated on a 4‐point Likert scale (0–3), yielding subscale scores from 0 to 21. Higher scores indicate greater symptom severity. A score of ≥ 8 suggests clinical anxiety or depression, with HADS‐A showing 0.90 sensitivity and 0.78 specificity, and HADS‐D 0.83 sensitivity and 0.79 specificity [28]. Scores are classified as normal (0–7), mild [8, 9, 10], moderate [11, 12, 13, 14], or severe [15, 16, 17, 18, 19, 20, 21].

#### 2.4.3 Measurement of Life Satisfaction

Life satisfaction was measured using the Satisfaction With Life Scale (SWLS), a 5‐item questionnaire rated on a 7‐point Likert scale (1 = strongly disagree, 7 = strongly agree) [29]. Total scores range from 5 to 35, with higher scores indicating greater satisfaction. Based on Pavot and Diener's (1993) guidelines, scores are categorized as follows: 31–35 (very satisfied), 26–30 (satisfied), 21–25 (slightly satisfied), 20 (neutral), 15–19 (slightly dissatisfied), 10–14 (dissatisfied), and 5–9 (very dissatisfied) [29].

#### 2.4.4 Measurement of Self‐Esteem

The Rosenberg Self‐Esteem Scale assesses global self‐worth using 10 items rated on a 4‐point Likert scale (1 = strongly agree to 4 = strongly disagree) [30]. Items 2, 5, 6, 8, and 9 are reverse‐scored, as they are negatively worded. Total scores range from 10 to 40, with higher scores indicating greater self‐esteem [30]. Based on total scores, participants are classified as having high [30, 31, 32, 33, 34, 35, 36, 37, 38, 39, 40], medium [26, 27, 28, 29], or low (< 26) self‐esteem. Medium scores suggest the need for some improvement, while low scores indicate significant problems [30].

#### 2.4.5 Measurement of Fear of Movement

Fear of movement was assessed using the Tampa Kinesiophobia Scale (TKS‐11) [31]. Participants were asked to assess their level of agreement with 11 items from 1 (totally disagree) to 4 (totally agree) [31]. Total scores ranged from 11 to 44 points, with higher scores indicating greater fear of pain, movement, and injury [31]. The intensity of fear could be categorized into: absence of fear (≤ 17 points), slight fear (18–24 points), moderate fear (25–31 points), severe fear (32–38 points), and maximum fear (39–44 points) [32].

#### 2.4.6 Measurement of Weekly Physical Activity

Physical activity was assessed using the Spanish version of the International Physical Activity Questionnaire (IPAQ) [33, 34]. Participants reported the number of days and minutes per day spent walking, performing moderate‐intensity activities, and performing vigorous‐intensity activities during the previous 7 days. From these data, the total weekly minutes of physical activity were calculated. The Spanish version used in this study was validated by Roman‐Viñas et al. [34], showing good reliability for total physical activity (*r* = 0.82, *p* < 0.05), vigorous activity (*r* = 0.79, *p* < 0.05), moderate activity (*r* = 0.83, *p* < 0.05), and walking time (*r* = 0.73, *p* < 0.05).

### 2.5 Statistical Analysis

Statistical analyses were conducted using IBM SPSS Statistics (Version 28). Repeated measures ANOVA was applied to compare pre‐ and post‐intervention data across all participants who received the intervention in each group (HRVG, PEG, CG), after normalizing variables using the two‐step transformation method [35]. Bonferroni post‐hoc tests were used for pairwise comparisons. Partial eta squared (ηp^2^) was calculated to assess the variance explained by each predictor. Clinical changes were assessed by counting participants who shifted levels in fatigue, pain, anxiety, depression, self‐esteem, life satisfaction, and fear of movement, based on validated cut‐off points. Pearson correlations were used to perform exploratory associations between variable changes within each group, following normalization and without Bonferroni correction due to the exploratory character and small sample size in each group to prevent Type II errors and potential significant interactions [36, 37]. Statistical significance was set at *p* < 0.05.

## 3 Results

### 3.1 Participants' Characteristics

**FIGURE 2:**
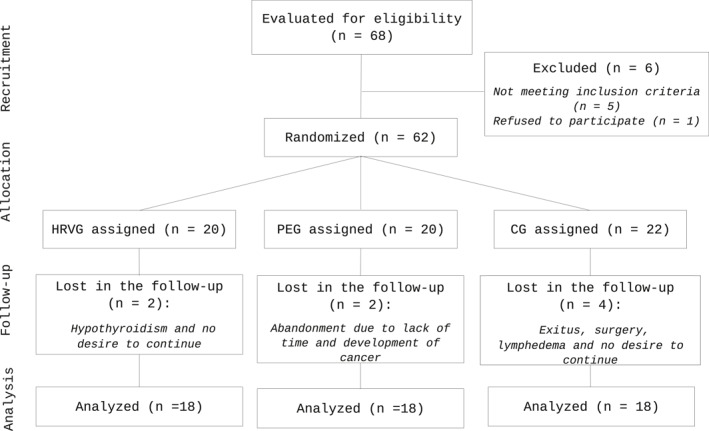
Flow diagram of the participants following CONSORT guidelines.

Of the 68 participants evaluated for eligibility, 62 started the intervention, and 54 completed the study (18 in each group). Dropout reasons are presented in Figure 2. Of the 5 participants excluded for not meeting eligibility criteria, 2 were excluded due to lack of a smartphone, 2 due to scheduling conflicts with the exercise program, and 1 due to cardiac abnormalities, according to the combined cardiac evaluation and criteria of the hospital's oncology and cardiology teams. The small number of participants excluded for smartphone access suggests that this criterion did not meaningfully affect the generalizability of the study findings.

### 3.2 Effects on Health‐Related Quality of Life

In the assessment of HRQoL, significant differences were observed in the overall health subscale between groups and evaluation times, with significant differences between CG and HRVG, as well as between CG and PEG. Intragroup differences revealed significant differences between pre‐ and post‐evaluation in all groups, increasing by 36.7% in HRVG and 15.61% in PEG, while decreasing by 20.34% in CG.

As shown in Table 2, physical, emotional, social, and role functioning showed significant differences across groups and time points, while cognitive functioning did not. Pairwise comparisons for physical functioning revealed significant intragroup changes in the control group (CG: −12.73%), HRV‐guided group (HRVG: +13.96%), and pre‐planned exercise group (PEG: +5.83%), with significant differences between CG–HRVG and PEG–CG. In role functioning, significant intragroup changes were observed only in HRVG, with differences between HRVG–CG and HRVG–PEG. For emotional functioning, both HRVG and CG showed significant intragroup changes, with improvements of 16.55% in HRVG and 10.51% in PEG. Significant group differences were noted between HRVG–CG and PEG–CG. Social functioning improved significantly within both HRVG and PEG, with increases of 33.8% and 22.53%, respectively.

**TABLE 2:** Descriptive and interaction results of global health and functional HRQoL dimensions of effects of exercise and usual care groups.

Variable	HRV group mean (SD)		PE group mean (SD)		Control group mean (SD)		ANOVA Time*group *p*‐value (ES)	Between‐group	Within group
	Baseline	After	Baseline	After	Baseline	After			
Global health	4.39 (0.50)	6.00 (0.62)	4.81 (1.11)	5.56 (0.87)	4.92 (1.23)	3.92 (1.03)	**18.25; *p* < 0.001 (0.417)**	**HRVG‐CG: *p* < 0.001** **HRVG‐PEG: *p* = 0.008** **PEG‐CG: *p* < 0.001**	**HRVG: *p* < 0.001** **PEG: *p* = 0.001** **CG: *p* = 0.002**
Physical functioning	82.22 (13.53)	93.70 (11.08)	88.89 (9.70)	94.07 (7.88)	86.67 (13.33)	75.29 (18.07)	**6.36; *p* = 0.004 (0.206)**	**HRVG‐CG: *p* < 0.001** HRVG‐PEG: *p* = 0.092 **PEG‐CG: *p* = 0.006**	**HRVG: *p* = 0.004** **PEG: *p* = 0.042** **CG: *p* = 0.008**
Role functioning	78.70 (22.00)	93.52 (8.36)	86.11 (20.81)	89.81 (15.27)	79.63 (23.26)	70.59 (27.97)	**1.33; *p* = 0.005 (0.190)**	**HRVG‐CG: *p* < 0.001** **HRVG‐PEG: *p* = 0.040** PEG‐CG: *p* = 0.137	**HRVG: *p* = 0.002** PEG: *p* = 0.357 CG: *p* = 0.234
Emotional functioning	74.07 (24.23)	83.33 (24.25)	75.46 (22.59)	83.39 (24.72)	74.54 (27.63)	62.25 (28.89)	**4.76; *p* = 0.013 (0.161)**	**HRVG‐CG: *p* < 0.001** HRVG‐PEG: *p* = 0.760 **PEG‐CG: *p* < 0.001**	**HRVG: *p* = 0.028** PEG: *p* = 0.072 **CG: *p* = 0.006**
Cognitive functioning	70.37 (25.28)	81.48 (27.35)	77.78 (19.80)	77.83 (25.39)	75.00 (34.42)	68.63 (29.98)	2.82; *p* = 0.085 (0.092)	**HRVG‐CG: *p* = 0.004** HRVG‐PEG: *p* = 0.302 PEG‐CG: *p* = 0.069	HRVG: *p* = 0.066 PEG: *p* = 0.282 CG: *p* = 0.166
Social functioning	65.74 (20.98)	87.96 (25.35)	71.30 (28.47)	85.29 (17.56)	70.37 (32.11)	65.69 (30.88)	**17.94; *p* = 0.001 (0.234)**	HRVG‐ CG: *p* = 0.740 HRVG‐PEG: *p* = 1.00 PEG‐CG: *p* = 0.676	**HRVG: *p* < 0.001** **PEG: *p* = 0.002** CG: *p* = 0.544

*Note:* Bold font: *p*‐value < 0.05.

Abbreviations: ES: Effect size; HRV: Heart rate variability; PE: Pre‐planned; SD: Standard deviation.

Significant differences were found between groups and over time (pre–post) in fatigue, pain, dyspnea, insomnia, and constipation, but not in appetite loss, diarrhea, nausea, or financial difficulties (*p* > 0.05). Pairwise comparisons revealed that the HRVG group showed significant intragroup improvements in fatigue (−50.81%), pain (−64.10%), dyspnea, and insomnia. In the PEG group, only pain improved significantly (−37.14%). In contrast, the CG group experienced a significant 37.49% increase in fatigue. Detailed results are presented in Table 3.

**TABLE 3:** Descriptive and interaction results of HRQoL symptoms dimensions of effects of exercise and usual care groups.

Variable	HRV group mean (SD)		PE group mean (SD)		Control group mean (SD)		ANOVA Time*group *p*‐value (ES)	Between‐group	Within group
	Baseline	After	Baseline	After	Baseline	After			
Fatigue	37.65 (25.32)	18.52 (24.70)	30.86 (28.66)	23.46 (23.15)	34.57 (28.49)	47.53 (24.34)	**18.34; *p* < 0.001 (0.413)**	**HRVG‐CG: *p* < 0.001** HRVG‐PEG: *p* = 0.082 **PEG‐CG: *p* = 0.002**	**HRVG: *p* = 0.001** PEG: *p* = 0.065 **CG: *p* = 0.004**
Nausea	0.93 (3.96)	0.00 (0.00)	0.00 (0.00)	0.00 (0.00)	0.93 (3.93)	0.93 (3.93)	1.047; *p* = 0.374 (0.038)	HRVG‐CG: *p* = 0.220 HRVG‐PEG: *p* = 0.221 PEG‐CG: *p* = 1.000	HRVG: *p* = 0.316 PEG: *p* = 1.00 CG: *p* = 1.00
Pain	36.11 (29.29)	12.96 (24.64)	32.41 (23.20)	20.37 (19.43)	36.11 (27.56)	40.74 (38.87)	**12.57; *p* < 0.001 (0.331)**	**HRVG‐CG: *p* < 0.001** HRVG‐PEG: *p* = 0.185 **PEG‐CG: *p* = 0.008**	**HRVG: *p* < 0.001** **PEG: *p* = 0.034** CG: *p* = 0.282
Dyspnea	24.07 (25.06)	9.26 (25.06)	18.52 (26.13)	7.41 (18.28)	20.37 (23.26)	31.48 (15.71)	**6.14; *p* = 0.004 (0.194)**	**HRVG‐CG: *p* < 0.001** HRVG‐PEG: *p* = 0.405 **PEG‐CG: *p* = 0.011**	**HRVG: *p* = 0.012** PEG: *p* = 0.053 CG: *p* = 0.058
Insomnia	42.59 (33.96)	25.93 (31.43)	35.19 (31.25)	24.07 (27.55)	33.33 (34.30)	42.59 (29.83)	**4.79; *p* = 0.012 (0.158)**	**HRVG‐CG: *p* = 0.009** HRVG‐PEG: *p* = 0.782 ***P*EG‐CG: *p* = 0.020**	**HRVG: *p* = 0.045** PEG: *p* = 0.055 CG: *p* = 0.096
Appetite loss	20.37 (25.92)	5.56 (12.78)	1.85 (7.86)	1.85 (7.86)	16.67 (30.78)	20.37 (34.56)	**2.717;** *p* = 0.082 (0.093)	**HRVG‐CG: *p* = 0.005** **HRVG‐PEG: *p* = 0.023** PEG‐CG: *p* = 0.647	**HRVG: *p* = 0.010** PEG: *p* = 1.00 CG: *p* = 0.608
Constipation	22.22 (28.01)	9.26 (19.15)	14.81 (23.49)	11.11 (16.17)	29.63 (39.42)	40.74 (38.87)	**4.68; *p* = 0.014 (0.155)**	**HRVG‐CG: *p* = 0.004** HRVG‐PEG: *p* = 0.304 PEG‐CG: *p* = 0.066	HRVG: *p* = 0.053 PEG: *p* = 0.527 CG: *p* = 0.034
Diarrhea	0.00 (0.00)	5.56 (23.57)	7.41 (14.26)	1.85 (7.86)	0.00 (0.00)	3.70 (15.71)	**2.056**; *p* = 0.148 (0.072)	HRVG‐CG: *p* = 0.981 **HRVG‐PEG: *p* = 0.027** **PEG‐CG: *p* = 0.030**	HRVG: *p* = 0.314 PEG: *p* = 0.082 CG: *p* = 0.157
Financial difficulties	9.26 (15.36)	7.41 (18.28)	5.56 (12.78)	9.26 (19.15)	29.63 (34.09)	33.33 (36.16)	0.707; *p* = 0.511 (0.026)	HRVG‐CG: *p* = 0.278 HRVG‐PEG: *p* = 0.288 PEG‐CG: *p* = 0.982	HRVG: *p* = 0.680 PEG: *p* = 0.317 CG: *p* = 0.157

*Note:* Bold font: *p*‐value < 0.05.

Abbreviations: ES: Effect size; HRV: Heart rate variability; PE: Pre‐planned; SD: Standard deviation.

Clinical changes in fatigue and pain are illustrated in Supporting Information S1: Figure S1, showing the number of participants in each group with scores above and below the clinical cut‐off at baseline and post‐intervention.

### 3.3 Effects on Anxiety and Depression

As shown in Table 4, significant differences in anxiety and depression were observed between groups and over time. Post hoc analyses revealed significant within‐group improvements in the HRVG for both anxiety (*p* = 0.001) and depression (*p* = 0.005). In the CG, depression worsened significantly (*p* = 0.002). Figure S2 illustrates that both HRV‐guided and pre‐planned groups improved, with more participants reaching normal anxiety and depression levels, especially in the HRVG. In contrast, the control group showed increased severity across both variables.

**TABLE 4:** Mental health and weekly minutes of physical activity descriptive and interaction results of effects of exercise and usual care groups.

Variable	HRV group mean (SD)		PE group mean (SD)		Control group mean (SD)		ANOVA Time*group *p*‐value (ES)	Between‐group	Within group
	Baseline	After	Baseline	After	Baseline	After			
Anxiety	8.33 (4.53)	4.83 (3.65)	7.56 (3.82)	6.22 (3.21)	7.17 (5.04)	8.00 (4.35)	**8.81; *p* = 0.002 (0.257)**	**HRVG‐CG: *p* < 0.001** HRVG‐PEG: *p* = 0.052 PEG‐CG: *p* = 0.061	**HRVG: *p* = 0.001** PEG: *p* = 0.086 CG: *p* = 0.230
Depression	5.78 (4.53)	2.89 (2.91)	4.00 (3.43)	3.33 (1.18)	4.33 (5.18)	6.17 (5.07)	**12.93; *p* < 0.001 (0.336)**	**HRVG‐CG: *p* < 0.001** HRVG‐PEG: *p* = 0.080 **PEG‐CG: *p* = 0.003**	**HRVG: *p* = 0.005** PEG: *p* = 0.117 **CG: *p* = 0.002**
Life satisfaction	20.22 (5.95)	27.72 (5.33)	22.22 (12.78)	26.11 (5.30)	22.78 (6.61)	18.94 (4.99)	**7.22; *p* = 0.003 (0.221)**	**HRVG‐CG: *p* < 0.001** HRVG‐PEG: *p* = 0.055 **PEG‐CG: *p* < 0.001**	**HRVG: *p* < 0.001** **PEG: *p* = 0.002** **CG: *p* = 0.003**
Self‐esteem	19.33 (3.09)	24.72 (3.56)	22.56 (5.94)	24.78 (4.43)	22.22 (4.36)8	21.22 (2.88)	**4.7; *p* = 0.023 (0.156)**	HRVG‐ CG: *p* = 1.00 HRVG‐PEG: *p* = 0.576 PEG‐CG: *p* = 0.369	**HRVG: *p* < 0.001** **PEG: *p* = 0.010** CG: *p* = 0.236
Fear of movement	29.61 (6.08)	22.72 (5.39)	26.17 (8.40)	22.67 (7. 19)	27.50 (8.35)	29.11 (8.19)	**5.6; *p* = 0.015 (0.180)**	HRVG‐ CG: *p* = 1.00 HRVG‐PEG: *p* = 1.000 PEG‐CG: *p* = 0.304	**HRVG: *p* < 0.001** **PEG: *p* = 0.002** CG: *p* = 0.143
Physical activity (minutes/week)	175 (71.50)	771. 67 (492.92)	130 (94.1)	693.61 (331.74)	147.22 (78.7)	347.78 (273.87)	**7.34; *p* = 0.002 (0.224)**	**HRVG‐ CG: *p* = 0.007** HRVG‐PEG: *p* = 1.00 PEG‐CG:*p* = 0.073	**HRVG: *p* < 0.001** **PEG: *p* < 0.001** **CG: *p* = 0.017**

*Note:* Bold font: *p*‐value < 0.05.

Abbreviations: ES: Effect size; HRV: Heart rate variability; PE: Pre‐planned; SD: Standard deviation.

### 3.4 Effects on Life Satisfaction, Self‐Esteem and Fear of Movement

Results for life satisfaction, self‐esteem, and fear of movement (Table 4) showed significant differences across time and groups. Life satisfaction improved by 37.1% in HRVG (*p* < 0.001) and 17.51% in PEG (*p* = 0.002), while CG declined by 16.86% (*p* = 0.003). Between‐group comparisons showed significant differences between CG and both HRVG and PEG (*p* < 0.001). Self‐esteem increased by 27.88% in HRVG (*p* < 0.001) and 9.84% in PEG (*p* = 0.01). Fear of movement decreased by 23.27% in HRVG (*p* < 0.001) and 13.37% in PEG (*p* = 0.002). Figure S3 illustrates that HRVG showed the greatest improvements across all measures. PEG also improved, while CG declined in all three variables.

### 3.5 Effects on Weekly Physical Activity

Significant differences were obtained in the interaction time and group minutes of physical activity per week. Although all groups significantly increased their physical activity level (HRVG: *p* < 0.001; PEG: *p* < 0.01; CG: *p* = 0.017), differences between groups were only shown between the HRVG and the CG.

### 3.6 The Relationship Between Psychological Parameters Regarding the Effects of Different Exercise Programs

**FIGURE 3:**
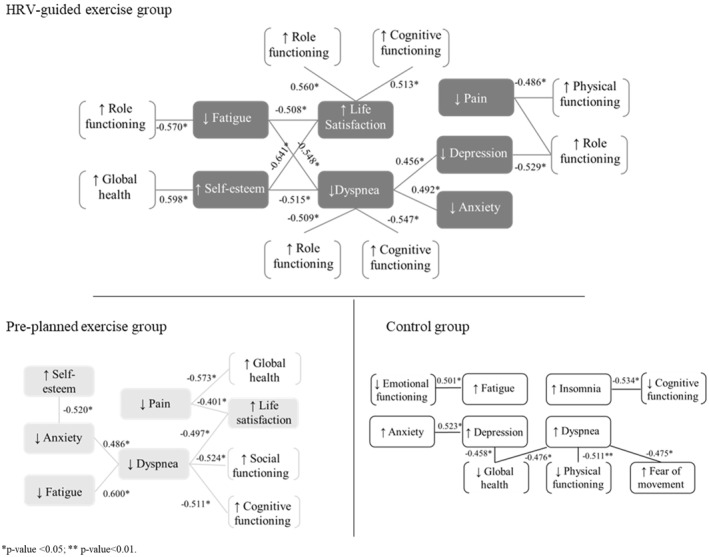
Pearson correlations of the changes in the psychological, functional and symptoms variables.

Pearson correlations (presented in Figure 3 and in Supporting Information S1: Table S1 of the supporting information) revealed in this exploratory analysis that HRV‐guided exercise might have more significant associations than PEG or CG. Exact *p*‐values for all correlations are reported in Supporting Information S1: Table S1 to allow better and more complete interpretation. In the HRVG, reductions in fatigue seemed to be associated with improvements in role functioning and life satisfaction, and might also relate to changes in dyspnea. Gains in self‐esteem may suggest a relationship with higher life satisfaction and global health, while also appearing inversely related to dyspnea. Increases in dyspnea might be linked to higher levels of depression, anxiety, and pain, as well as lower role and cognitive functioning. Changes in pain seemed inversely related to physical and role functioning, which in turn may also show associations with depression.

In the PEG, fatigue appeared to correlate positively with dyspnea. Dyspnea might be associated with anxiety and fatigue, and may show inverse relationships with cognitive and social functioning, as well as life satisfaction. Changes in pain seemed negatively associated with global health and life satisfaction, while anxiety might be related to self‐esteem.

As for the CG, dyspnea may be inversely associated with global health and physical functioning, and could show a positive association with fear of movement. Global health seemed inversely related to depression, and fatigue might be linked to lower emotional functioning.

## 4 Discussion

The purpose of this research was to analyze the effects of online supervised high‐intensity interval training and high‐load strength training, prescribed based on daily individual HRV, compared to pre‐planned interval and strength training of moderate to high intensity/load and usual care on different psychological variables that influence the mental health of breast cancer survivors.

In terms of HRQoL, both intervention groups improved in physical and social functioning, but only physical functioning differed significantly from the control group, highlighting the general value of exercise for supporting daily activities in cancer survivors. However, role and emotional functioning improved only with HRV‐guided training, offering greater benefits for managing work, hobbies, and emotional symptoms like anxiety and depression. The control group showed a decline in emotional functioning. Cognitive functioning showed no significant change, possibly due to the absence of a specific cognitive intervention [38]. Regarding symptoms, HRV‐guided exercise significantly improved fatigue, pain, dyspnea, appetite loss, and insomnia, while pre‐planned training improved only pain. Fatigue, a major concern in this population [39], decreased by 50.81% in the HRV group but rose by 37.49% in controls. Patients with higher fatigue often show reduced HRV, reflecting autonomic imbalance and inflammation [16]. Lower RMSSD scores were linked to greater fatigue, suggesting rMSSD‐based exercise may better manage fatigue and related symptoms.

After the completion of in‐hospital treatments, women with hormone‐dependent cancers typically continue adjuvant therapy with oral medications and/or injections for at least 5 years. This prolonged therapy may contribute to the persistence or even exacerbation of certain symptoms or side effects from previous treatments, such as fatigue and pain [40]. This trend is evident in the control group findings of the present study, in which, consistent with other research, women who continued with the usual care reduced their overall health, physical and emotional function, and satisfaction with life, increasing their fatigue and depression [3]. Diarrhea, nausea, and financial difficulties did not show significant improvement with any of the programs, which was expected, as patients were not under chemotherapy.

In terms of emotional symptoms, the HRVG significantly improved anxiety and depression between times and in comparison to the control group in both variables. These results suggest that personalizing exercise based on individual HRV may be more effective than standardized programs. Prior research indicates that anxiety mediates the link between sympathetic activation and quality of life and that exercise helps regulate mood disorders [41]. Physiologically, exercise promotes neurogenesis and cognitive function by releasing chemicals like beta‐endorphins, serotonin, vascular endothelial growth factor, and brain‐derived neurotrophic factor [42]. It also influences the hypothalamic‐pituitary‐adrenal axis, reducing anxiety by lowering cortisol levels and improving hormonal regulation through increased endorphin production and serotonergic system changes [43].

In line with these findings, Li et al. (2024) reported reduced anxiety and depression through online aerobic programs in cancer patients [44]. Similarly, Courneya et al. (2014) found that higher volumes of exercise during chemotherapy were particularly effective for breast cancer patients with significant depressive symptoms [45]. In our study, only the group performing higher intensity and volume of exercise showed significant reductions in both anxiety and depression, shifting from severe to more normal levels. This suggests that increased exercise intensity, even delivered online, can improve mental health outcomes through both biological and psychosocial mechanisms. Given that over 30% of women with breast cancer experience depression [46], these results are socially significant. Moreover, anxiety and depression can worsen cancer‐related symptoms such as fatigue, highlighting the importance of addressing mental health in this population [39].

Findings on life satisfaction, self‐esteem, and fear of movement suggest that both pre‐planned and HRV‐modulated exercise can significantly improve these psychological variables. However, personalized approaches showed greater percentage changes and clinically meaningful improvements. Although less studied than quality of life or anxiety, these factors are crucial for understanding individual differences and barriers to exercise participation, as seen in other health conditions [47]. In breast cancer, for example, fear of movement may hinder exercise initiation and adherence due to concerns about injury, lymphedema, fatigue, or joint pain, all of which affect exercise self‐efficacy [47]. Similarly, low self‐esteem, often stemming from physical changes post‐treatment or low perceived competence [48], may make exercising in shared spaces challenging, especially when surrounded by individuals without similar experiences. Understanding these psychological barriers is essential for designing effective, inclusive exercise programs. Careful planning, clear explanations, tailored correction, and supportive feedback can help foster safety, confidence, and sustained engagement in physical activity for women with breast cancer [18].

The associations between variables revealed exploratory patterns, indicating that the highest degree of connectivity between changes might be observed in the HRV‐guided group. Network theory in mental health suggests that symptoms are interrelated and influence one another [49]. Applying this approach to exercise interventions in cancer patients could help identify central symptoms and their impact. For example, exercise‐related reductions in pain, reported by 48.17% of this population [50], appeared to occur together with increases in overall health in the pre‐planned group and with higher role and physical function in the HRVG group. Better physical condition tended to coincide with lower pain levels and increases in overall well‐being, a significant aspect given that high pain levels are associated with discontinuation of hormone therapy [51]. Similarly, variations in dyspnea and fatigue seem to be associated with differences in depression scores, functionality, and cognitive function, whereas unfavorable changes in these symptoms might be related to declines in health measures in the usual care group. Focusing on these interconnected symptoms through exercise‐based strategies could be important for understanding quality of life dynamics among breast cancer survivors.

Although the benefits of physical exercise are well‐studied, few investigations have examined supervised online exercise programs, and none have achieved the present level of success [21]. This may be due to the real‐time supervision provided in all sessions, which ensures proper execution, adherence, and safe progression of high‐intensity exercise, while also promoting social interaction, self‐efficacy, and self‐esteem, further supporting mental health [18]. Integrating technology and personalized approaches can enhance adherence and outcomes by enabling specific monitoring of HRV, exercise frequency, and intensity. This allows for tailored exercise prescriptions, real‐time feedback, and communication between professionals and patients, enhancing confidence and control [52]. This enhances confidence and a sense of control in participants, as trainers can personalize intensity based on feedback about recovery, fatigue, stress, or discomfort, ensuring the program meets their needs. However, success may depend on participants' willingness to use the technology, access to devices, and trainers' ability to implement and explain the tools. Combining these technologies with adherence strategies like the PADEX scale [19], as done in this study, can improve supervision, support, and outcomes, especially in managing chronic diseases.

### 4.1 Clinical Implications

This study represents a clinical advance by implementing, for the first time, parasympathetic modulation as an objective tool to adjust training intensity based on recovery and fatigue in female breast cancer survivors. This methodology allows for safer and more personalised exercise prescription, reducing the potential risks associated with overload and increasing the effectiveness of exercise, as the significant results in most of the psychological variables showed. From a research perspective, future lines of study are opening about its application in different training contexts and the analysis of the interaction between autonomic control and psychological factors, to enhance the effectiveness of exercise‐based clinical interventions, which are very necessary to contrast the findings.

## 5 Limitations

This study has some limitations. First, it included women with both triple‐negative and luminal breast cancer, leading to varied prior treatments that may affect their perceptions of quality of life and psychological factors when beginning the exercise program. Second, only women with luminal cancer were on hormone therapy during the study, making side effects like fatigue and pain more noticeable. Third, the small sample size prevented subgroup analysis by severity level. Moreover, the within‐group sample size was insufficient to support robust association analyses; therefore, these results should be considered exploratory and secondary to the main interaction analysis, without implying a causal relationship. Future research should explore how exercise benefits vary with initial levels of anxiety, depression, self‐esteem, life satisfaction, or fear of movement.

## 6 Conclusion

This study shows that online supervised concurrent exercise improves HRQoL and psychological outcomes in breast cancer survivors. Both personalized HRV‐guided high‐intensity training and pre‐planned moderate to high‐intensity exercise enhanced self‐esteem, life satisfaction, and reduced fear of movement. However, the HRV‐guided group experienced greater improvements in physical, emotional, and global health, along with significant reductions in pain, fatigue, and dyspnea. Individualized training also led to larger decreases in anxiety and depression. Conversely, the control group showed declines in HRQoL, depression, and life satisfaction. These results highlight the benefits of personalized exercise based on parasympathetic modulation, supporting its role in recovery and quality of life, and call for further research on tailored exercise interventions for breast cancer survivors.

## Author Contributions

A.M.L.‐P. and D.C.‐M. wrote the original manuscript. I.N., X.M., C.H.G., A.d.J.F., G.L., and A.J. revised the manuscript. A.M.L.‐P., D.C.‐M., and X.M. analyzed and interpreted the data. A.M.L.‐P., C.H.G., A.d.J.F., G.L., and A.J. conceptualized the study and defined the methods. A.M.L.‐P. collected the data. I.N. and G.L. were responsible for data curation. All authors approved the final version of the manuscript.

## Ethics Statement

The study adhered to the ethical standards outlined in the Declaration of Helsinki. Ethical approval was obtained from the Ethics Committee of King Juan Carlos University (reference number 1901202103121) and from the Ethics Committee of the Hospital Universitario Marqués de Valdecilla, affiliated with the Valdecilla Health Research Institute (IDIVAL), under registration number 42//2021. All participants provided informed consent prior to their inclusion in the study.

## Conflicts of Interest

The authors declare no conflicts of interest.

## Supporting information


Supporting Information S1

## Data Availability

The data that support the findings of this study are available from the corresponding author upon reasonable request.
