# Supplementary material for: Effects of Individualized High‐Intensity Online Concurrent Exercise Guided by Autonomic Modulation on the Mental Health and Quality of Life of Breast Cancer Survivors
Source: Psychooncology. 2025 Dec 3;34(12):e70348. doi: 10.1002/pon.70348 (PMC12674982; doi:10.1002/pon.70348)
Supplement: Supplementary file 1 — Supporting Information S1 [file PON-34-e70348-s001.docx]

**Supporting Information**


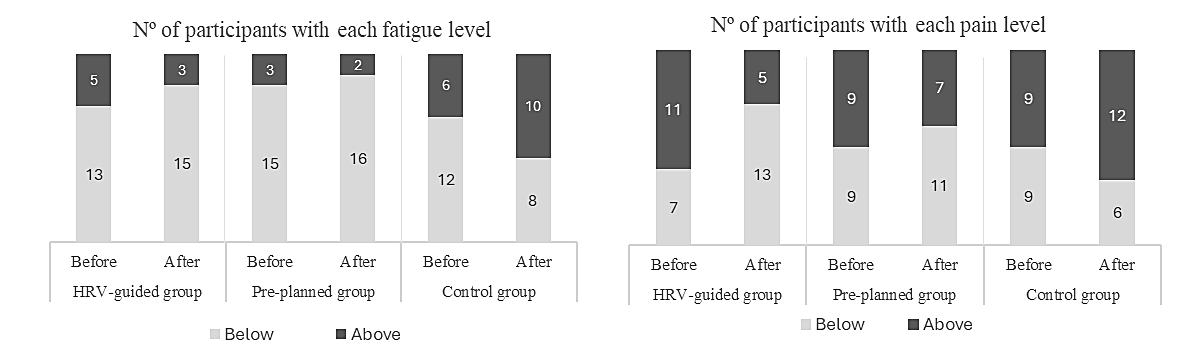
**Figure S1**. Changes in the number of participants with below and above cutoff levels of fatigue and pain.


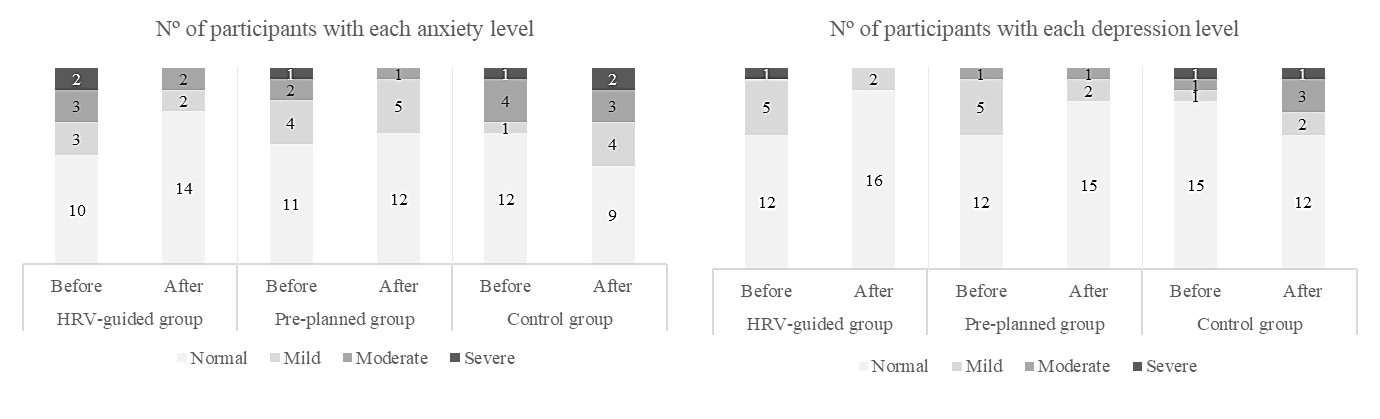
**Figure S2**. Changes in the number of participants with normal, mild, moderate and severe levels of anxiety and depression.


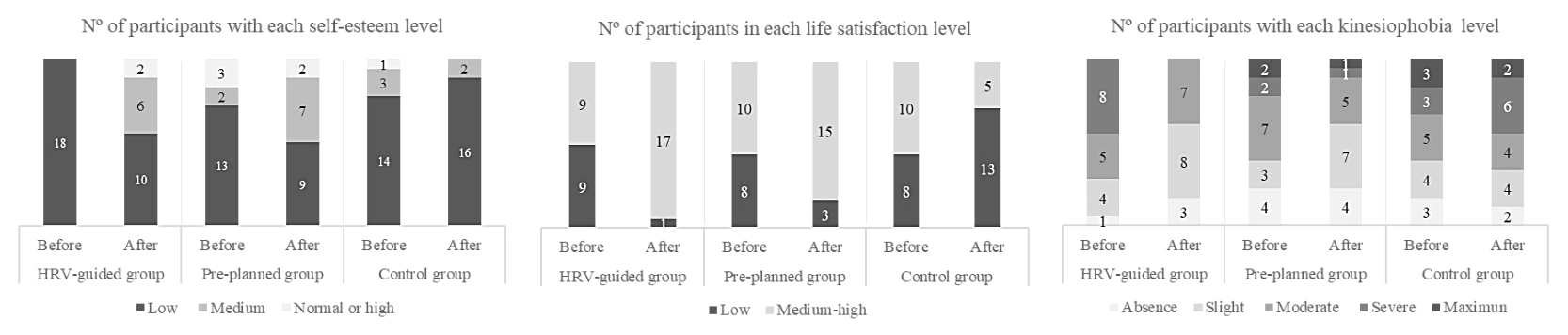
**Figure S3**. Changes in the number of participants with each level of self-esteem, life satisfaction and kinesiophobia.

**Table S1**. Pearson correlations of the changes in psychological, functional, and symptom-related variables across study groups.

| **HRV-Guided Group** | | | **Pre-planned Group** | | | **Control Group** | | | |
| --- | --- | --- | --- | --- | --- | --- | --- | --- | --- |
| **Significant correlations** | **r** | **p-value** | **Significant correlations** | **r** | **p-value** | | **Significant correlations** | **r** | **p-value** |
| Fatigue – Role Function | -0.570 | 0.0137 | Self-esteem – Anxiety | -0.520 | 0.0277 | | Fatigue – Emotional Function | 0.501 | 0.0348 |
| Fatigue – Life Satisfaction | -0.508 | 0.0320 | Anxiety – Dyspnea | 0.486 | 0.0429 | | Anxiety – Depression | 0.523 | 0.0271 |
| Fatigue – Dyspnea | -0.548 | 0.0192 | Fatigue – Dyspnea | 0.600 | 0.0077 | | Depression – Global Health | -0.458 | 0.0486 |
| Self-esteem – Dyspnea | -0.515 | 0.0291 | Pain – Global Health | -0.573 | 0.0125 | | Dyspnea – Physical Function | -0.511 | 0.0318 |
| Self-esteem – Global Health | 0.598 | 0.0082 | Pain – Life Satisfaction | -0.401 | 0.0497 | | Dyspnea – Fear of Movement | -0.475 | 0.0468 |
| Life Satisfaction – Role Function | 0.560 | 0.0155 | Dyspnea – Social Function | -0.524 | 0.0264 | | Insomnia – Cognitive Function | -0.534 | 0.0231 |
| Life Satisfaction – Cognitive Function | 0.513 | 0.0300 | Dyspnea – Life Satisfaction | -0.497 | 0.0369 | | — | — | — |
| Physical Function – Pain | -0.486 | 0.0428 | Dyspnea – Cognitive Function | -0.511 | 0.0316 | | — | — | — |
| Depression – Role Function | -0.529 | 0.0240 | — | — | — | | — | — | — |
| Dyspnea – Anxiety | 0.492 | 0.0398 | — | — | — | | — | — | — |
| Dyspnea – Cognitive Function | -0.547 | 0.0195 | — | — | — | | — | — | — |
| Dyspnea – Role Function | -0.509 | 0.0313 | — | — | — | | — | — | — |
